# An Accumulated Activity Effective Index for Promoting Physical Activity: A Design and Development Study in a Mobile and Pervasive Health Context

**DOI:** 10.2196/resprot.3336

**Published:** 2015-01-06

**Authors:** Chung-Tse Liu, Chia-Tai Chan

**Affiliations:** ^1^Department of Biomedical EngineeringNational Yang-Ming UniversityTaipeiTaiwan

**Keywords:** accumulated activity effective index (AAEI), physical activity, activity level

## Abstract

**Background:**

Increased physical activity has become a principal personal health goal worldwide because sufficient physical activity can reduce the risk of many adverse conditions. Motivating individuals to increase their levels of physical activity can increase life expectancy and contribute to a healthy life. Sharing and comparison of physical activity information by using the Internet, with fewer privacy concerns, might also help encourage people to promote and maintain sufficient physical activity. To promote and manage physical activity, an accumulated activity effective index (AAEI) is proposed in this paper.

**Objective:**

The purpose of the AAEI design is to maintain and promote physical activity. The public can easily accept a clear indicator that reveals the current status of physical activity. The AAEI is not only an assessment and tracking tool for personal physical activity, but is also useful for goal setting and for sharing content with the Internet community.

**Methods:**

The AAEI is derived from input in the form of accumulated physical activity, and evaluates the status of physical activities and days spent exercising. The term AAEI(t_1_,t_2_) is an index of the accumulated physical activity in the time interval (t1,t2), where the base unit of time is the day. The AAEI is determined according to accumulated physical activity and is adjusted using the previous status of physical activity. The previous status of physical activity is estimated according to the number of days spent exercising and the accumulated physical activity that has been performed. An analysis of the AAEI performance was conducted using a simulation model and a real-world trial with 2 participants.

**Results:**

The AAEI increased as the physical activity and days spent exercising accumulated. Conversely, the AAEI decreased with lack of physical activity and increased resting days. In simulation, the shape of the AAEI line indicated different types of exercise. The moving average AAEI represented long-term exercise. In the real-world trial, the AAEI confirmed that the simulation results were comparable to actual conditions.

**Conclusions:**

The AAEI proposed in this paper is a method that can be used to evaluate the status of a person’s physical activity. The AAEI is a simple numeric indication that is estimated by analyzing accumulated physical activity and the average number of days spent exercising. The AAEI is suitable for tracking personal physical activity, reminding the user of achievement goals, and allows data sharing by using the Internet. The results have demonstrated that the AAEI is a useful tool for physical activity management.

## Introduction

Sufficient physical activity has substantial benefits for a healthy life. Regular and moderate intensity of physical activity, such as fast walking, running, and cycling, can reduce the risk of coronary heart disease, type 2 diabetes mellitus, and depression, as well as facilitate weight control [1-3]. Moreover, physical activity increases bodily health and improves cognitive functioning. It increases resistance to neurodegenerative diseases, dementia, and related cognitive impairments [4]. Unfortunately, 31.1% of adults worldwide are physically inactive. Physical inactivity increases with age and is more prevalent in high-income countries [1]. According to previous studies, physical inactivity is the fourth leading risk factor for mortality and noncommunicable diseases, and caused 5.3 million deaths worldwide in 2008 [5]. Physical inactivity increases the risk of many adverse health conditions and threatens global health. The elimination of physical inactivity can increase life expectancy and contribute to a healthy life.

Pervasive computing technologies are well suited for health care applications and have the potential to promote a healthy lifestyle. Several well-known studies have been proposed using pervasive computing technologies to assist individuals in achieving sufficient physical activity [3,6-9]. For example, the pedometer can become a human activity sensor used to monitor physical activity and promote health because walking is a health-boosting activity and a pedometer can help motivate and track progress. Although the accuracy of pedometers can be unreliable, they have been shown to motivate individuals toward a more active lifestyle [8-10]. The progress of measuring instruments has allowed multiple sensing modules to be built which can provide information such as blood pressure and heart rate. This information is critical in health care applications; however, the professional terms and complicated interface can confuse the public and can be a barrier to the popularization model for usage of such instruments; for the public, a simple indicator is easier to accept.

Motivating individuals to increase their levels of physical activity is a critical issue in health promotion. Numerous studies have focused on the social aspects revealing that the sharing and comparison of information regarding physical activity within the community can increase interest in, and enjoyment of, exercise and can motivate people to be more active [7,9,11]. The high penetration of Internet and community websites can enhance communication in groups and can be used as a medium to motivate physical activity. However, personal context information such as time, location, and heart rate when shared using the Internet can suffer personal privacy problems. Other studies suggest that goal setting can increase self-regulatory behaviors and improve physical activity [12]. An accumulated activity effective index (AAEI) is proposed in this paper for evaluating the status of physical activity and sharing related information with communities on the Internet. The AAEI is designed to provide a simple numeric indication of accumulated physical activity and days spent exercising, with fewer privacy concerns than personal context information. The AAEI is designed to increase awareness of physical activity by tracking physical activity, reminding the user of achievement goals, and sharing this information with the Internet community. The AAEI is also suited for self-awareness in maintaining physical activity. The performance of the AAEI was illustrated using a simulation model and a short-term real-world trial using pervasive computing tools.

## Methods

### Accumulated Activity Effective Index

**Figure 1:**
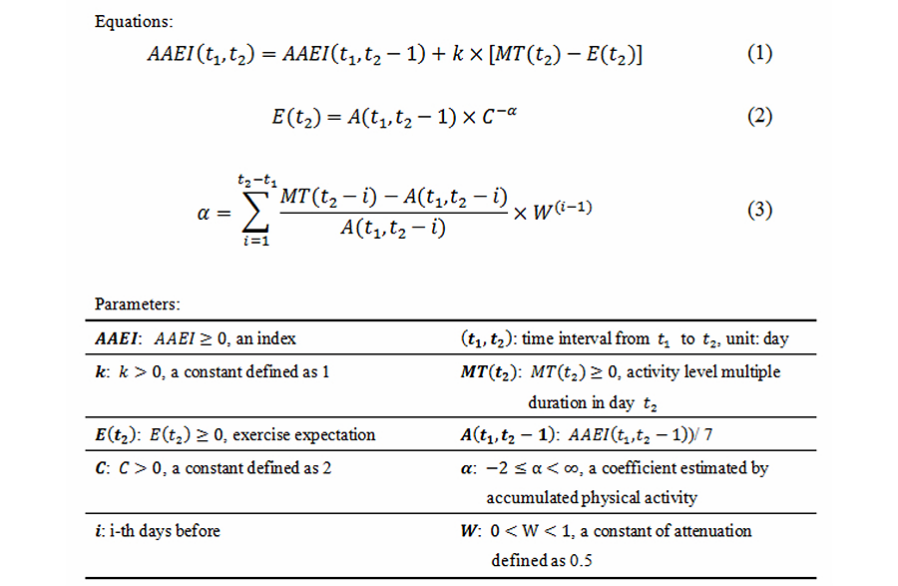
The accumulated activity effective index (AAEI) parameters and equations for the evaluating process (equation 1) and exercise expectance (equations 2 and 3).

The AAEI was designed as a simple numeral indicator that directly reveals the physical activity status of the user by estimating both accumulated physical activity and days spent exercising. The design principles of the AAEI were (1) AAEI is a simple value to reveal physical activity; (2) AAEI corresponds to physical activity; (3) AAEI increases with more physical activity, is steady in fixed physical activity, and decreases with less physical activity; (4) AAEI corresponds to days spent exercising; (5) AAEI decreases with resting days; (6) AAEI decreases more with continued resting; (7) AAEI decreases less at rest if user has exercised before; and (8) AAEI is at or near zero if the user does not exercise in 7 days. The AAEI parameters and evaluating process (Equation 1) are described in Figure 1.

The term AAEI(t_1_,t_2_) is an index of the accumulated physical activity in the time interval (t_1_,t_2_), where the base unit of time is the day. The AAEI is greater than or equal to zero. The AAEI(t_1_,t_2_) is calculated by tracking the sum of AAEI(t_1_,t_2_–1) and the amount of physical activity in t_2_. The variable MT(t_2_) represents the amount of physical activity in t_2_, which is equal to the activity level multiplied by the exercise duration. The variable E(t_2_) is defined as the exercise expectation of physical activity in t_2_, which depends on the previous interval (t_1_,t_2_–1) status of physical activity include accumulated physical activity and days spent exercise. Parameter k is a constant value and is greater than zero to scale the AAEI. The design of k in a different constant or a variable can be a condition depending on AAEI(t_1_,t_2_–1), MT(t_2_), or others. For example, k can increase with activity level, in which case participants exercise harder and can get a higher AAEI because AAEI increases more during vigorous activity. In another example, if k rises when [MT(t_2_)–E(t_2_)]<0, the user needs to sustain physical activity to maintain AAEI.

The previous status of physical activity was defined as *exercise expectance,* which is greater than or equal to zero and was formulated using Equations 2 and 3 in Figure 1. The notion of exercise expectation, E(t_2_), was that if a participant had a high AAEI, a participant was expected to require more physical activity to increase the AAEI. Otherwise, AAEI would be stable or decrease. Another design objective for exercise expectation was to reveal days spent exercising. The more days a participant rested, the more the AAEI index decreased. The variable A(t_1_,t_2_–1) is the basic value defined as AAEI(t_1_,t_2_–1)/7. The variable C is a constant greater than zero, which adjusts the decreasing percentage of resting. In the design principle, no decreasing is defined when there is no resting. In other words, if a participant exercises every day, the AAEI does not decrease. Increasing constant C decreases the average AAEI, but does not increase the AAEI at the same amount of accumulated physical activity. Parameter α is a coefficient determined by previous accumulated physical activity. The variable W is a constant of attenuation that decreases the influence of previous physical activity. Thus, the greater the value of W, the more the previous accumulated physical activity and days spent exercising affect the exercise expectance. To achieve convergence, the absolute value of W should be less than 1 in Equation 3 in Figure 1. To receive the positive AAEI, the multiplication of k and C to the power of the summation of W to the power of (i-1), where i goes from 1 to (t_2_-t_1_) should be less or equal to 7. In this study, for example, we set W at 0.5 causing the influence of previous accumulated physical activity to decrease 50% after 1 day. The variable t indicates the day—zero for today, 1 for yesterday, and so forth. According to the aforementioned conditions, the range of α is between –2 and infinity, and the range of E(t_2_) is between A(t_1_,t_2_–1)×C^2^ and zero. The initial condition of the AAEI evaluation process to be set was AAEI(t_1_,t_2_)=0, E(t_2_)=0, and α was null because there was no physical activity recorded before. The AAEI is useful for evaluation purposes from the first time a person performs physical activity. Because A(t_1_,t_2_–i) is null the first time to estimate AAEI, parameter α is calculated the first time after physical activity is recorded. In our prototype experiment, the defined parameters k=1, W=0.5, and C=2 are used to examine the performance of the AAEI.

### Simulation Model

A simulation model was used to test the long-term performance of the AAEI. Based on the assumption adopted in previous analyses of human dynamics, human behavior comprises temporal statics, which are uniform and stationary. In other words, most human activities can be described using a Poisson process [13-14]. Based on these characteristics, the simulation model simulated participants partaking in exercise of various exercise durations, and the average activity level for each exercise had a Poisson distribution. The distribution of days of the week on which participants exercised was random. Different types of exercise habits were included to simulate the performance of the AAEI.

### Real-World Trial

**Figure 2:**
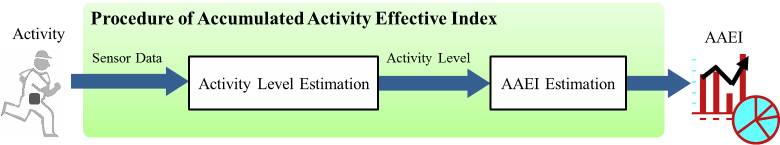
The process in a real-world trial. The first stage was activity level estimation. The second stage was AAEI estimation. The sensor data translated to activity level in first stage and then converted to AAEI in second stage.

Two participants were recruited to test the AAEI performance in a real-world application. Before the AAEI evaluation, participants estimated their activity levels and exercise duration as inputs of physical activity evaluation process, as shown in Figure 2. Several methodologies were developed to estimate physical activity, such as calorimetry, double-labeled water, questionnaires, and wearable sensors. This study used a triaxial accelerometer (Opal, APDM Inc, Portland, OR, USA) to estimate activity levels because it is inexpensive, accurate, small, objective, sensitive, and suitable for the storing of personal records. Several studies have estimated activity levels by using accelerometers [15-17]. The activity level estimation methodology employed in this study was designed based on that of Liu et al [15]; the experimental results of this study showed high accuracy levels of approximately 80%. The sampling rate of the triaxial accelerometer was 40 Hz. The sensor was worn on the right side of the front of the waist. To reduce any deviation in the estimation of activity level, the exercise tasks were limited to walking, fast walking, and running, and were all performed using a running machine at various velocities. All participants were allowed to perform the tasks within their discretion during the 1-month trial period. The AAEI was estimated once per day.

## Results

### General Results

**Figure 3:**
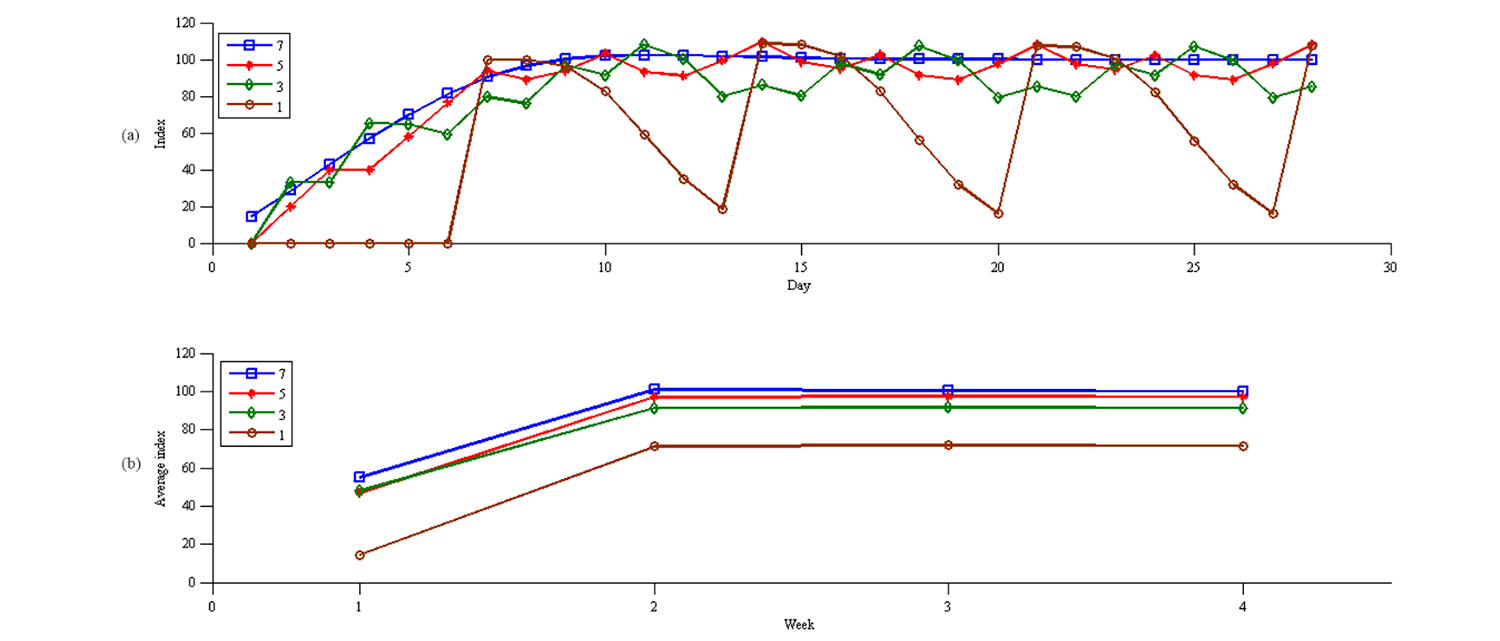
(a) AAEI of ideal and periodic physical activity input and (b) week average of AAEI.

**Figure 4:**
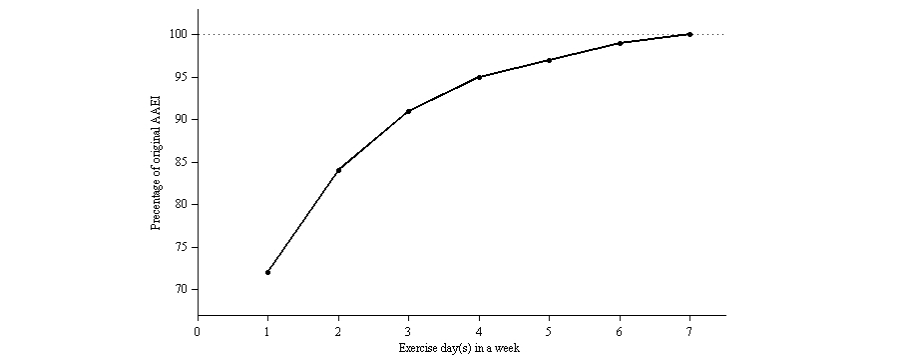
Average percentage of AAEI for different exercise days per week in an ideal situation.

The ideal amount and period of physical activity was used as input to illustrate the general performance of the AAEI. A total of 100 metabolic equivalent of task (MET)-minutes were evenly distributed over 1, 3, 5, and 7 day(s) in a week (Table 1) over a period of 4 weeks as shown in Figure 3. The AAEI increased when physical activity increased, did not increase when physical activity was stable, decreased when physical activity decreased, and decreased further if continuous resting occurred. The weekly average of the AAEI over the 4 weeks is shown in Figure 3. The AAEI continued to increase in the first week, but became stable after the second week. The *i*-day (where *i*=1, 3, 5, 7) averages of the stable AAEI changed because of various resting days. The average percentage of the AAEI on different days of physical activity under stable conditions is presented in Figure 4.

**Table 1:** Input distribution of ideal and periodic physical activity in a week.

Exercise days per week	Input (MET-minutes)						
	Day 1	Day 2	Day 3	Day 4	Day 5	Day 6	Day 7
1	0	0	0	0	0	0	100
3	0	33.3	0	33.3	0	0	33.3
5	0	20	20	0	20	20	20
7	14.3	14.3	14.3	14.3	14.3	14.3	14.3

### Simulation Results

**Figure 5:**
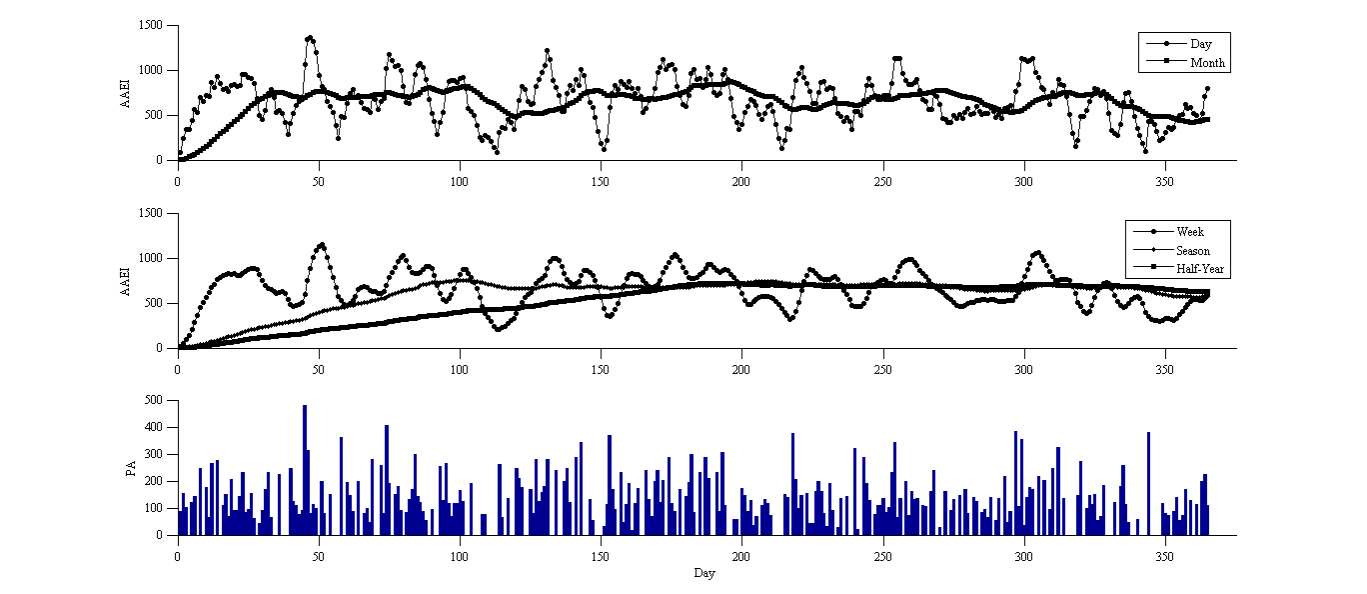
AAEI/PA simulation results with input of 5 days per week, duration of 30 minutes, and 5 MET.

Three simulation results are presented to show the long-term performance of the AAEI. Each simulation result included different types of physical activity input to present different exercise conditions. Figure 5 shows the exercise protocol proposed by the World Health Organization (WHO), which recommends exercising 5 days per week, each exercise having a 30-minute duration and 5 MET on average. These recommendations were used as parameters and entered into the simulation model. The simulation model generated an average of 718 MET-minutes per week and 4.8 days spent exercising per week over a year. The average AAEI was 663 for 1 year. The ideal ratio of AAEI to physical activity (ideal AAEI/PA), as shown in Figure 4, was 0.97. The ratio of AAEI to physical activity (AAEI/PA) in the simulation was 0.92. The day line of the AAEI went up and down based on the average AAEI value. The longer-term moving averages showed smoother line changes than the average AAEI value.

**Figure 6:**
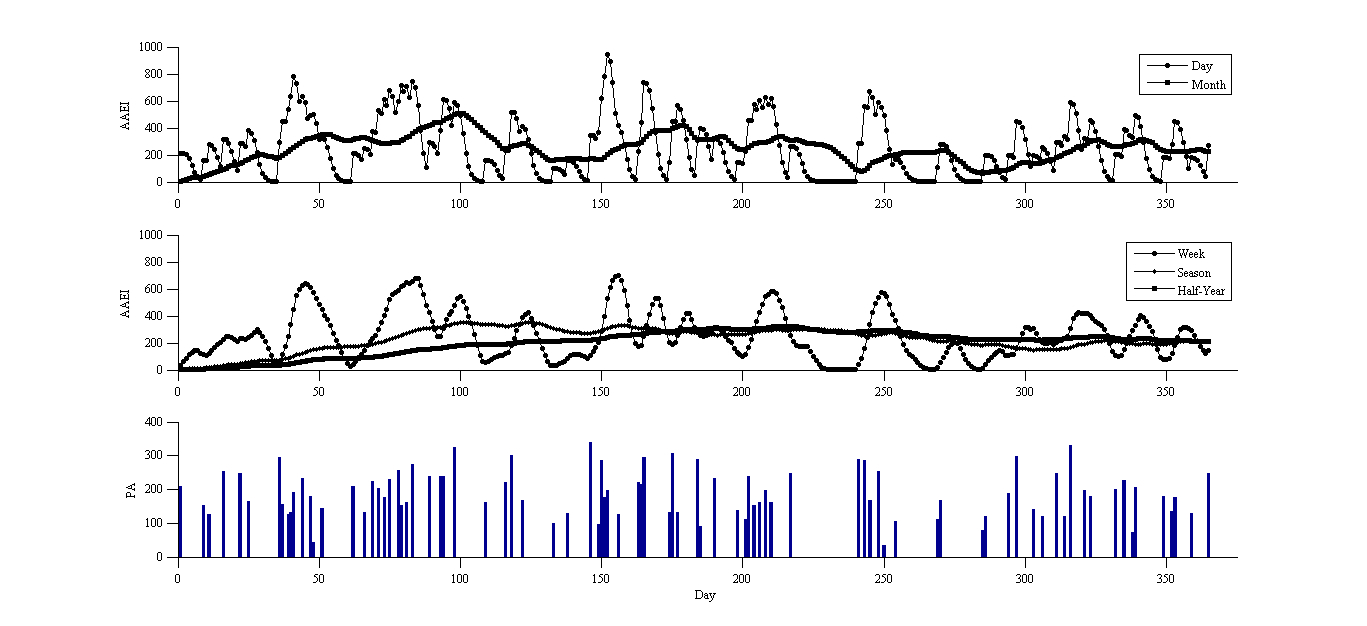
AAEI/PA simulation results with input of 2 days per week, duration of 20 minutes, and 10 MET.

Some people perform vigorous physical activity, but only for a short time interval. To simulate this condition, the simulation model set 2 days of exercise per week, each with an average duration of 20 minutes, and an average activity level of 10 MET. The simulation model generated 313 MET-minutes per week and 1.6 days spent exercising per week, on average, over a year. The average AAEI was 254. The ideal AAEI/PA was 0.80 and the AAEI/PA was 0.81. Figure 6 shows the results of the simulation and the AAEI estimate. The day line of the AAEI formed a peak when physical activity was performed and formed a valley when there was a lack of physical activity.

**Figure 7:**
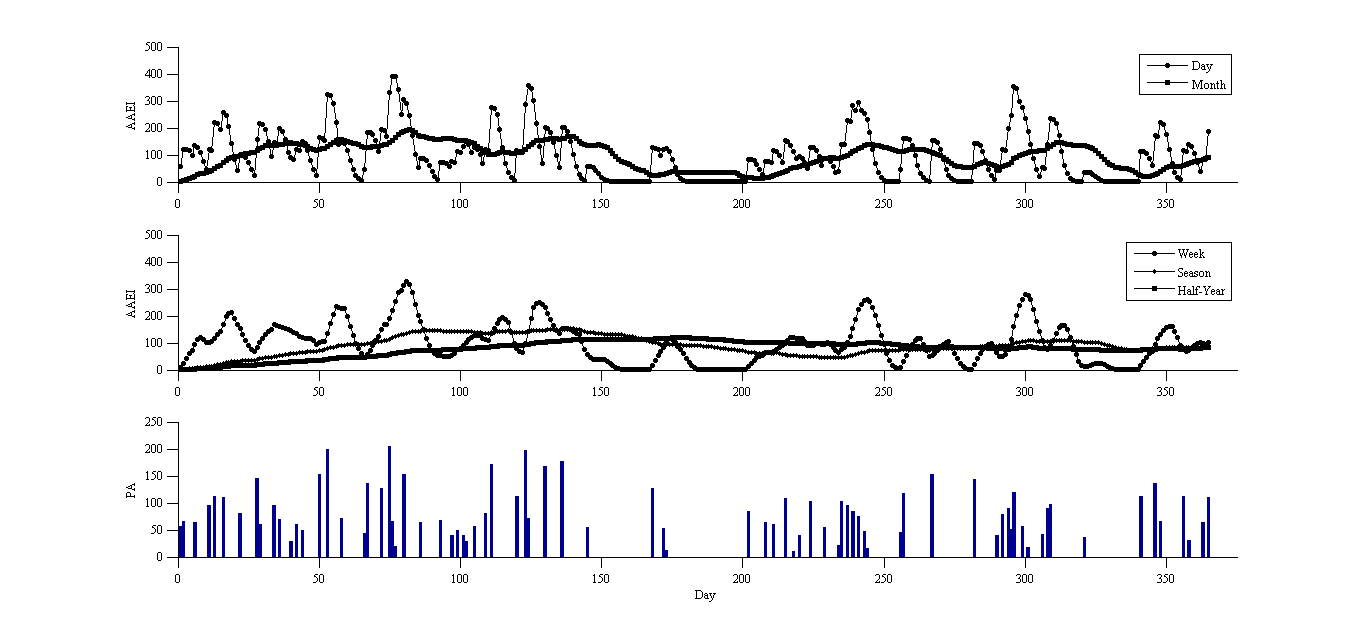
AAEI/PA simulation results with input of 2 days per week, duration of 15 minutes, and 5 MET.

To simulate physical inactivity, the simulation model was set at 2 days spent exercising per week, each with an average duration of 15 minutes and an average of 5 MET as parameters. The simulation model generated an average of 127 MET-minutes per week and 1.5 days spent exercising per week over a year. The average AAEI was 100. The ideal AAEI/PA was 0.78 and the AAEI/PA was 0.79. Figure 7 shows the simulated results; the day line of the AAEI formed a steep shape when continuous resting occurred and the AAEI line was often low and near the zero line.

### Real-World Trial Results

A total of 2 participants were recruited to take part in the real-world trial test. The participants’ characteristics are listed in Table 2. The recorded physical activity was measured according to observation and activity diaries. Estimated values were measured according to an accelerometer. Table 3 lists the recorded and estimated total physical activity.

**Table 2:** Participant characteristics (N=2).

Variables	Participant 1	Participant 2
Gender	Male	Male
Age (years)	24	23
Body mass index	21.4	22.3
Medical status	Healthy	Healthy

**Table 3:** Result of total recorded physical activity and total estimated physical activity.

Participant	Total recorded value (MET-minutes)	Total estimated value (MET-minutes)	Ratio
Participant 1	1211	1178	0.973
Participant 2	945	1031	1.057

**Figure 8:**
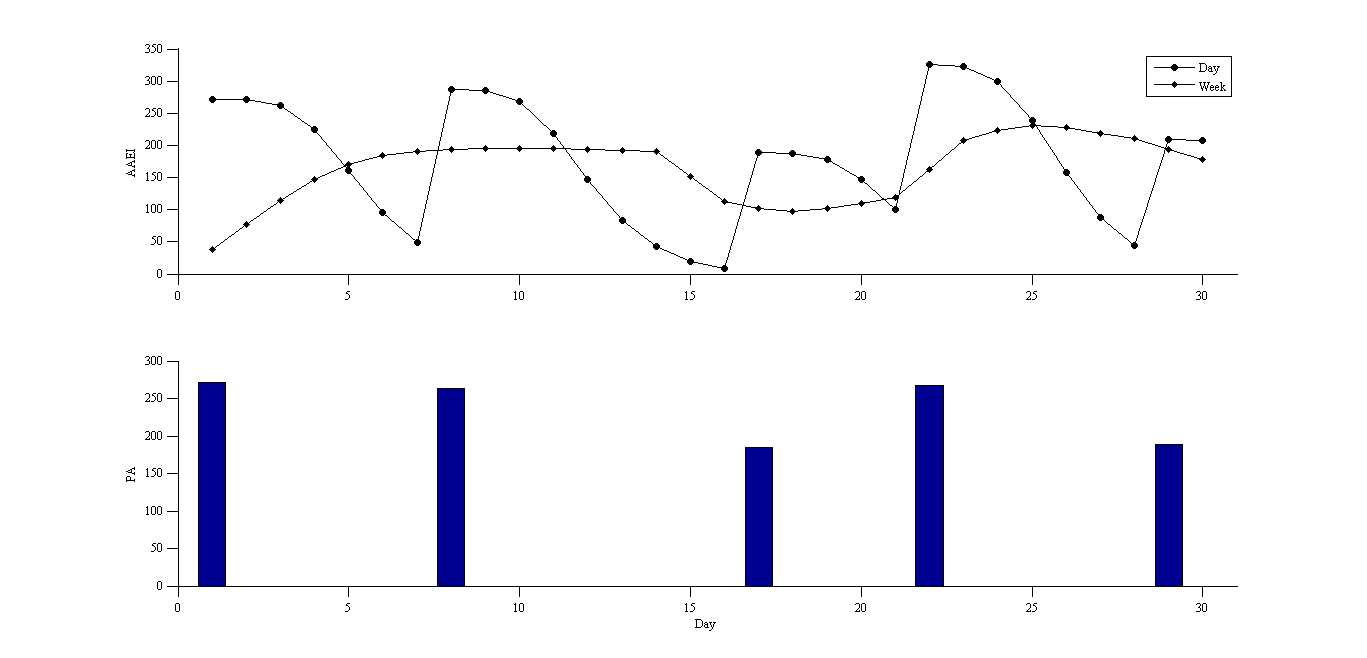
AAEI with physical activity (PA) of participant 1.

The average physical activity and days spent exercising shown in Figure 8 were 275 MET-minutes per week and 1.2 days per week, respectively. The average AAEI was 180, the ideal AAEI/PA ratio was 0.74, and the AAEI/PA was 0.65.

**Figure 9:**
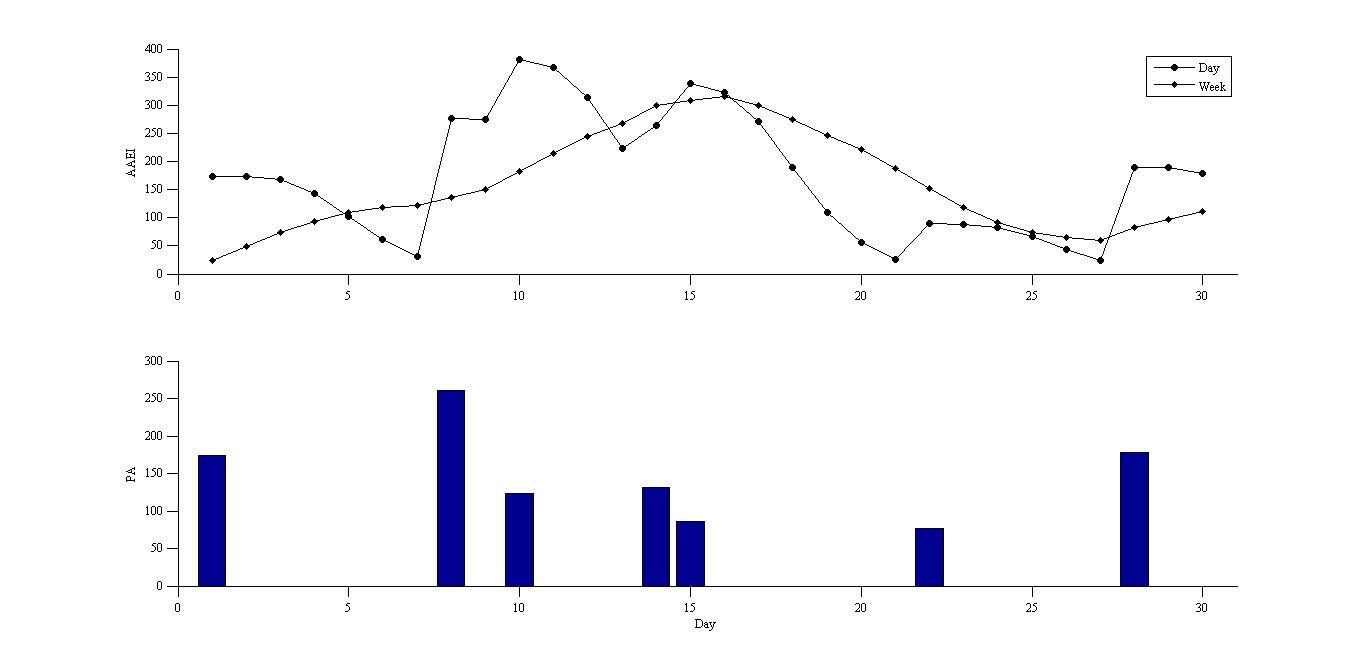
AAEI with physical activity (PA) of participant 2.

The average physical activity and days spent exercising shown in Figure 9 were 241 MET-minutes per week and 1.6 days per week, respectively. The average AAEI was 174, the ideal AAEI/PA ratio was 0.80, and the AAEI/PA was 0.72.

## Discussion

### General Performance

The AAEI was determined using the accumulated physical activity and exercise expectance. In our study, the AAEI increased when physical activity increased, did not increase when physical activity was stable, decreased when physical activity decreased, and decreased further if continuous resting occurred. The first week of ideal and periodic inputs increased because the AAEI accumulated from the first 7 days. After the first week, the AAEI stabilized because of stationary input. Although the amount of physical activity in a week was the same, the AAEI altered on different days spent exercising. The more days participants exercised, the higher the AAEI was. The AAEI was directly proportional to the amount of physical activity accumulated in 7 days. The AAEI was primarily determined using the accumulated amount of physical activity and associated with the number of days spent exercising. The value of the AAEI was decided by accumulated physical activity more than days spent exercising. Exercise expectance, estimated using the previous physical activity status, accumulated physical activity, and number of days spent exercising, would revise the AAEI. Thus, the index usually decreased further when AAEI was high. We encouraged participants whose physical activity was low to be more active, and challenged those who performed large amounts of physical activity. A higher AAEI typically indicated more physical activity and days spent exercising; therefore, the decrease in percentage was more reasonable than fixed reducing. The constant *C* is used to revise the magnification of exercise expectance. A higher *C* value decreased the AAEI further in response to physical inactivity, but increased the AAEI little in higher physical activity situations. The setting value of the constant *C* can influence the average AAEI in general. Parameter k was defined as a fixed number in this study and, therefore, the AAEI directly revealed the status of physical activity. The parameter k was set to 1 because it can directly reveal the accumulated amount of physical activity. However, the setting value of k can differ depending on the status of physical activity in the Web-based application because of encouragement or challenge. The variable W is a constant of attenuation. We set W at 0.5 in this experiment causing the effect of the previous status to attenuate by 50% after each day.

### Establishing a Simulation

The moving average AAEI across a range of durations revealed the varying physical activity statuses of the participants across different points in time. The long-term moving average AAEI varied slightly compared with the short-term moving average AAEI. When the short-term moving average AAEI line crossed the long-term line, it indicated that the physical activity of a participant was either higher or lower than before. We observed the index to ascertain whether the physical activity of the participant was increasing or decreasing. The simulation results reveal the phenomenon, but exhibited deviations in the amount by which physical activity decreased. Because the distribution of physical activity was not uniform in the simulation, the AAEI deviated from the estimate. The deviation of the AAEI was within 5% of the ideal situation. In the simulation model, in which the physical activity was the same as that recommended by the WHO, the achievement goal was an AAEI value of 600. If the participant reached the assigned index, he or she was likely to consider that sufficient physical activity had been performed. The other 2 simulation results exhibited insufficient physical activity in various exercise habits. The shape of the AAEI line demonstrates the different habits of the participants. A high and sharp peak of the AAEI line on the graph reveals that the participant partook in vigorous physical activity, but only over a short period. A flat and low shape of the AAEI line indicates the participant was physically inactive. The AAEI value in these 2 cases did not exceed 600; therefore, their physical activity was insufficient. An arc-shaped AAEI line with low, oscillating amplitude located at a high index (greater than 600) indicates that the participant partook in sufficient and regular physical activity. The AAEI can be used as an analysis tool for improving physical activity.

### Real-World Trial

The recorded physical activity was measured through observation as well as through analysis of activity diaries, and it generally agreed with the estimated value. Although the level of accuracy of the estimated value seems considerably high, the deviation was slightly compensated by overestimating and underestimating, but it still made it possible to evaluate the AAEI in a real-world application. Both participants were physically inactive in 1 month. Generally, the results of the AAEI demonstrated the physical activity status. The AAEI increased with increased physical activity and decreased with decreased physical activity. The AAEI decreased gradually, rather than suddenly. However, the deviation of decrease was much higher than in the simulation results, possibly because of nonuniform distribution in the short-term experiment. The AAEI did not immediately provide feedback to the participants and only showed the feasibility of real-world implementation of the AAEI. Based on previous studies [7,9,11], sharing and comparison can motivate people to be more active by increasing their interest in and enjoyment of physical activity. Goal-setting theory is based on the concept that people occasionally require a clearly defined goal to motivate them to achieve. The AAEI is not only an assessment and tracking tool for personal physical activity, but also a goal-setting and achievement-sharing tool in social contexts.

### Implementation of the Accumulated Activity Effective Index

Measurement of the level of activity and the energy cost was a fundamental task before the AAEI estimates were made. Therefore, the characteristics of the measurement of activity levels were crucial to the AAEI estimates because measurement of activity level can directly reveal participants’ physical activity. If the level of activity can be measured accurately, it can be useful in calculating the AAEI. One can apply preferred activity-monitoring devices and the proposed AAEI to measure physical activity for individual well-being management. Furthermore, the proposed AAEI is also useful for goal setting and for sharing content with the Internet community under the same criterion.

### Conclusions

The AAEI is proposed in this paper as a means of evaluating the status of physical activity. It can track personal physical activity, remind the user of his or her achievement goals, and share this information by using the Internet. The AAEI is a simple numeric indication that is estimated by accounting for accumulated physical activity and the average number of days spent exercising. The AAEI records the accumulated physical activity that has been performed in a week and reveals any differences in exercise habits. The moving average presents a long-term value that can be used for assessment purposes. The AAEI fulfills the requirement of prompting physical activity. Based on social aspect theory, the AAEI is a useful tool that can be employed to promote physical activity.

## Abbreviations

AAEI: accumulated activity effective index
MET: metabolic equivalent of task
PA: physical activity
WHO: World Health Organization
